# Ferroptosis, a new form of cell death, and its relationships with tumourous diseases

**DOI:** 10.1111/jcmm.13008

**Published:** 2016-11-10

**Authors:** Haitao Yu, Pengyi Guo, Xiaozai Xie, Yi Wang, Gang Chen

**Affiliations:** ^1^ Department of Hepatobiliary SurgeryThe First Affiliated HospitalWenzhou Medical UniversityWenzhouChina; ^2^ Environmental and Public Health School of Wenzhou Medical UniversityWenzhouChina

**Keywords:** ferroptosis, iron‐dependent cell death, tumourous diseases, erastin

## Abstract

Ferroptosis is a newly discovered type of cell death that differs from traditional apoptosis and necrosis and results from iron‐dependent lipid peroxide accumulation. Ferroptotic cell death is characterized by cytological changes, including cell volume shrinkage and increased mitochondrial membrane density. Ferroptosis can be induced by two classes of small‐molecule substances known as class 1 (system **X**_**c**_^**−**^ inhibitors) and class 2 ferroptosis inducers [glutathione peroxidase 4 (GPx4) inhibitors]. In addition to these small‐molecule substances, a number of drugs (e.g. sorafenib, artemisinin and its derivatives) can induce ferroptosis. Various factors, such as the mevalonate (MVA) and sulphur‐transfer pathways, play pivotal roles in the regulation of ferroptosis. Ferroptosis plays an unneglectable role in regulating the growth and proliferation of some types of tumour cells, such as lymphocytoma, ductal cell cancer of the pancreas, renal cell carcinoma (RCC) and hepatocellular carcinoma (HCC). Here, we will first introduce the discovery of and research pertaining to ferroptosis; then summarize the induction mechanisms and regulatory pathways of ferroptosis; and finally, further elucidate the roles of ferroptosis in human tumourous diseases.

## Introduction

A new form of cell death, ferroptosis, was recently discovered. Ferroptosis results from iron‐dependent lipid peroxide accumulation and is characterized mainly by cell volume shrinkage and increased mitochondrial membrane density without typical apoptotic and necrotic manifestations 1. Ferroptotic cell death can be induced by two classes of small‐molecule substances. Class 1 ferroptosis inducers include erastin, sulfasalazine (SAS), DPI2 and buthionine sulfoximine, which can inhibit system X_C_^−^ and reduce the intracellular glutathione content, causing an oxidation‐reduction imbalance in cells. Class 2 ferroptosis inducers include Ras selective lethal 3 compound (RSL3), DPI7, DPI10, DPI12, DPI13, etc., which can directly inhibit glutathione peroxidase 4 (GPx4) 2 and ultimately lead to an accumulation of lipid peroxides. In addition, ferroptosis can be induced by various drugs (e.g. sorafenib, artemisinin and its derivatives) 3, 4. The regulatory factors of ferroptosis primarily include the mevalonate (MVA) pathway, sulphur‐transfer pathways and the HSF1‐HSPB1 system 5. Ferroptosis may occur during a variety of physiological and pathological processes in humans and animals. Research has revealed the involvement of ferroptosis inhuman diseases. In particular, by regulating the growth and proliferation of tumour cells, ferroptosis plays an unneglectable role in the occurrence and progression of various tumourous diseases. To date, researchers have found that a number of tumour cell types, including lymphocytoma ductal cell cancer of the pancreas, renal cell carcinoma (RCC) and hepatocellular carcinoma (HCC) cells, are susceptible to ferroptosis 2, 6, 7. The goal of this review is to provide a general overview of current knowledge regarding the mechanisms underlying ferroptosis in cells, its role in the growth and proliferation of tumour cells, its relationship with human tumourous diseases, and the application of pro‐ferroptotic approaches in tumour treatment.

## Definition and discovery of ferroptosis

### What is ferroptosis?

Iron (Fe) is the fourth most common element in the Earth's crust, and it plays a pivotal role in human bodies 8. It is essential for cell survival because of its involvement in oxygen transportation, DNA biosynthesis and ATP synthesis as an auxiliary factor of various proteins in the tricarboxylic acid (TCA) cycle and the electron transport chain 9. In addition, iron has been found to be closely related to the occurrence and progression of tumours, and disorders of iron metabolism might facilitate tumour growth 9, 10. In addition, the presence of iron, particularly divalent iron, greatly accelerates lipid peroxidation of saturated fatty acids in humans 11. During iron‐involving oxidative phosphorylation in mitochondria, cells produce reactive oxygen species (ROS) along with the generation of ATP. ROS levels that exceed the cell's anti‐oxidation capacity can lead to an oxidative stress response, which directly and indirectly damages large molecular substances such as proteins, nucleic acids and lipids 12, leading to cell injury or death. This newly discovered form of cell death is called ferroptosis. Ferroptosis differs from apoptosis and necrosis in the traditional sense and results from the accumulation of iron‐dependent lipid peroxide 1.

### Discovery of ferroptosis

In the history of ferroptosis, ferroptosis inducers were actually discovered before ferroptosis was named. In a 2003 study using a large‐scale screening experiment to explore the killing effect of various chemical compounds on tumour cells, Stockwell *et al*. identified a new chemical compound, erastin, that can cause RAS‐mutated tumour cells to die in a manner different from traditional apoptosis 13. In 2008, Stockwell *et al*. discovered two new compounds, RSL3 and RSL5, that have the same effect as erastin. They also determined that the resulting cell death can be inhibited by an iron chelator, desferrioxamine B‐methane sulphonate (DFOM) and an antioxidant, vitamin E 14, confirming that this form of cell death is related to intracellular iron and ROS. In 2012, Stockwell *et al*. used the term ‘ferroptosis’ to describe this type of cell death caused by the accumulation of iron‐dependent lipid peroxides 1. Later, other chemical compounds, including sorafenib 15, artemisinins 4, 6, and a newly discovered five‐membered ring cyclic peroxide 1, 2‐dioxolane (FINO2) 16, were confirmed to have the ability to induce ferroptosis. Furthermore, the mechanisms underlying the induction of the key molecules, system X_C_^−^ and GPx4, in ferroptosis were partially revealed 2, 17. Recently, Xie *et al*. published a relatively detailed summary of ferroptosis inducers, inhibitors and regulatory molecules 18. However, a number of questions remain unaddressed, including the role of iron in ferroptosis, the molecular mechanisms underlying the induction of ferroptosis by ROS, and the roles of ferroptosis in human diseases.

## Differences between ferroptosis and apoptosis/necrosis

Cell death is the final stage of cells; it is caused by cytotoxicity from either exogenous or endogenous substances. There are various forms of cell death, which were originally defined and differentiated based on cellular morphology. In 1972, Kerr *et al*. defined a type of hepatatrophy‐associated ‘automatically programmed’ cell death as ‘apoptosis’ 30, which is characterized by typical morphological changes such as chromosome shrinkage, chromatin condensation and peripheralization, and round or oval cytoplasmic fragment formation 1, 30. Later, using an electron microscope, Schweichel and Merker observed the death of embryotic cells during their development in rats treated with/without lethal embryo toxicants and divided this programmed cell death (PCD) into three types 31. Clarke named type III PCD ‘necrosis’ 32, which is a passive form of cell death. As research has progressed, an increasing number of cell death types has been discovered, including pyroptosis, necroptosis, parthanatos, autophagy, oncosis and ferroptosis. Ferroptosis differs considerably from other cell death types, such as apoptosis, necrosis, and autophagy, in various aspects, including morphology, biochemistry and genetics 1, 14. Ferroptosis does not result in morphological changes similar to the chromatin condensation that occurs during apoptosis, the loss of plasma membrane integrity that occurs during necrosis, or the formation of double membrane‐layered autophagic vacuoles that occurs during autophagy; instead, it manifests primarily as mitochondrial shrinkage and increased mitochondrial membrane density 1 (Table 1).

**Table 1:** The main morphological features, regulators, inducers and inhibitors of ferroptosis, apoptosis, necroptosis and autophagy

Cell death	Defining morphological features	Regulators	Inducers	Inhibitors
Ferroptosis	Mitochondria become smaller, with increased mitochondrial membrane densities; reduced mitochondrial crista 1	Mitochondrial regulator genes: *RPL8, IREB2, CS, ATP5G3, TTC35, ACSF2* 1 Essential regulators: GPx4 Cancer cell regulators: P53, HSPB1, Rb, SLC7A11, VDACs, NRF2	Class1: erastin, erastin derivatives (MEII, PE, AE), DPI2, BSO, SAS, lanperisone, SRS13‐45, SRS13‐60 Class2: RSL3, DPI7, DPI10, DPI12, DPI13, DPI17, DPI18, DPI19, ML160 Drugs: sorafenib, artemisinin derivatives 10	Iron chelators: desferoxamine, solamine, 2, 2‐Bipyridyl Anti‐oxidants: vitamin E, U0126, Trolox ROS formation inhibitors: ferrostatin‐1, SRS8‐24, SRS8‐72, SRS11‐92, SRS12‐45, SRS13‐35, SRS13‐37, SRS16‐86, CA‐1 Others: cycloheximide, aminooxyacetic acid, ebselen, β‐mercaptoethanol 10
Apoptosis	Plasma membrane blebbing; cellular and nuclear volumereduction; nuclearfragmentation 19, 20	Apoptosis‐related genes: Proapoptotic: *CASP10, CARD8, GZMB* Antiapoptotic: *HSPA1B, CARD6, NOX5* 21 Core regulators: p53, Bax, Bak, Bcl‐2, Bcl‐XL	Extrinsic apoptosis: FASL, DCC, UNC5B Intrinsic apoptosis: multiple intracellular stress conditions (e.g. DNA damage, cytosolic Ca2+ overload) 19	IAPs: XIAP, c‐IAP1, c‐IAP2, ILP‐2, ML‐IAP/livin, NAIP, Bruce/Apollon, survivin 22
Necroptosis	Plasma membrane rupture; organelle swelling; moderate chromatin condensation 19, 20	Sensitive genes: *EDD1, MPG, CA9, SLC25A15, SIRT5, NPEPL1, DCC1, CD40 and COL4A3BP* 23 Core regulators: RIP1, RIP3, MLKL	TNFα zVAD.fmk 24, 25	RIP1 inhibitors: necrostatin1 (Nec‐1) 26 MLKL inhibitors: necrosulfonamide (NSA)
Autophagy	Formation of double‐membraned autolysosomes 19, 20	Regulator genes: *ATG genes (ATG1, 3, 4, 5, 6, 7, 8, 9, etc*.) 27 Core regulators: Beclin 1, ATG family proteins	Rapamycin, lithium, sodium, valproate, carbamazepine 28	Non‐selective PI3K inhibitors: 3‐ME, LY294002, wortmannin Selective VPS34 inhibitors: PIK‐III, compound 31, SAR 405, Vps34‐In1 Specific ULK1 inhibitors: MRT68921, MRT67307, SBI‐0206965 Specific Beclin1 inhibitors: Spautin‐1 Lysosome inhibitors: chloroquine, hydrochloroquin 29

ROS, reactive oxygen species; RSL, Ras selective lethal 3 compound; SAS, sulfasalazine; VDACs, voltage‐dependent anion channels.

Ferroptosis can be induced by various types of small molecules, such as erastin, SAS and RSL3; however, it cannot be induced by the substances that induce apoptosis and necrosis (e.g. N‐benzyloxycarbonyl‐Val‐Ala‐Asp‐fluoromethylketone (Z‐VAD‐FMK), Boc‐Asp (OMe)‐fluoromethylketone (Boc‐D‐FMK), wortmannin and necrostatin‐1) 1, suggesting that the mechanism underlying ferroptosis induction differs from that of apoptosis and necrosis (Table 1). In addition, compared with other fatal substances, ferroptosis‐inducing small molecules exhibit a remarkable selectivity towards cell strains 33. Moreover, the ferroptosis inducers generally differ from the inducers of other newly discovered cell death, forms such as necroptosis and pyroptosis 34.

Six mitochondrial genes, *RPL8, IREB2, ATP5G3, CS, TTC35* and *ACSF2* are involved in the genetic regulation of ferroptosis. Research has confirmed that these six genes are closely associated with ferroptosis but are irrelevant to other forms of cell death, such as apoptosis, necrosis or autophagy 1, suggesting that the genetic regulatory mechanism of ferroptosis is completely different from that of apoptosis and necrosis (Table 1). However, a recent study discovered that ferroptosis and ‘autophagy’, another type of cell death that differs from apoptosis and necrosis, share some common mechanisms, as evidenced by a finding that autophagy inhibitors and lysosomal activation can suppress ferroptosis by reducing the generation of cytoplasmic and lipid peroxides 35. Hence, the relationship of ferroptosis with other cell death types remains to be clarified, calling for further research and exploration.

## Mechanisms of ferroptosis

### Inhibition of system X_c_^−^leads to ferroptosis

System X_C_^−^ is a membrane Na+‐dependent cysteine‐glutamate exchange transporter, which is a disulphide‐linked heterodimer composed of a light‐chain subunit (xCT, SLC7A11) and a heavy‐chain subunit (CD98hc, SLC3A2) 36. While it transports intracellular glutamate to the extracellular space, system X_C_^−^ transports extracellular cystine into the cell 37, which is then transformed into cysteine for glutathione (GSH) synthesis. Cellular uptake of cysteine is a key step of GSH synthesis, and GSH generation and maintenance is critical for protecting cells from the damage caused by oxidative stress responses. System X_C_^−^ inhibition leads to a compensatory transcriptional upregulation of SLC7A11 in cells. Similar to erastin, SAS, another system X_C_^−^ inhibitor, can also act on HT‐1080 fibrosarcoma cells to induce an upregulation of SLC7A11 expression in cells 38. In addition, a study using 14C‐labelled cysteine found that treatment with erastin, SAS, or glutamate could lead to ferroptosis of HT‐1080 cells by remarkably reducing their ability to uptake cysteine and synthesize GSH. This effect could be inhibited by β‐mercaptoethanol (β‐ME) 1, because β‐ME can enhance cysteine uptake through other pathways 39. These findings further proved the involvement of system X_C_^−^ in ferroptosis triggered by the above mentioned inducers. Cells with erastin‐ or SAS‐induced ferroptosis have a significantly lower GSH level 2, which causes iron‐ and ROS‐dependent cell death by disrupting the oxidation‐ reduction balance in cells.

In the central nervous system (CNS), the neurotoxicity of glutamate is oxidative iron‐dependent 40, 41. Glutamate neurotoxicity can be inhibited by iron chelators and ferr‐1 1, implying a possible involvement in ferroptosis 40. Previous research revealed that glutamate toxicity results from either the calcium influx caused by the activation of glutamate receptors 42 or the inhibition of system X_C_^−^ by its competitive inhibitors 37, 43. However, Wolpaw *et al*. found that calcium chelators did not affect ferroptosis 44, indicating that the activation of glutamate receptors is not involved in ferroptosis. This finding indirectly proved the close relationship between system X_C_^−^ and ferroptosis.

### Direct inhibition of GPx4 leads to ferroptosis

By suppressing system X_C_^−^ to prevent extracellular cysteine from moving into cells and to reduce the intracellular GSH level, erastin results in iron‐dependent cell death mediated by the accumulation of lipid ROS. However, research has found that anti‐oxidants, including diethyldithiocarbamic acid [DETC, an inhibitor of superoxide dismutase (SOD)], diamide (DIA, a thiol‐reactive reagent) and 1‐chloro‐2, 4‐dinitrobenzene (DCNB, a thioredoxin reductase inhibitor), cannon‐selectively kill human foreskin fibroblast (BJeLR) cells without depleting the intracellular GSH pool 2. This suggests that erastin‐induced cell death does not necessarily result from only the suppression of the anti‐oxidative system. Researchers believe that instead, ferroptosis results from the effect of inducers on a specific downstream site of GSH. GPx4 was originally considered an inhibitory protein of lipid peroxidation 45 because it degrades H2O2 and other common small‐molecule peroxides and complex lipid peroxides 46. GPx4 is an enzyme that decomposes H_2_O_2_ and organic H_2_O_2_ into water or corresponding alcohols, and GSH is an essential cofactor in its activation 47. Therefore, by depleting the intracellular GSH pool, the ferroptosis inducers erastin and BSO reduce GPx4 activity and elevate cytoplasmic and lipid ROS levels 2, ultimately leading to cell ferroptosis. The GPxs family consists of various members, including GPx1‐8 48, and GPx4 plays a more important role than the others in ferroptosis 2. (1S, 3R)‐RSL is a ferroptosis inducer 14 that can directly bind to GPx4 and inhibit its activity 2, leading to the intracellular accumulation of lipid peroxides and subsequent ferroptosis. In addition to erastin and RSL3, other 12 ferroptosis inducers have been discovered in a large number of screening experiments 49, 50. Eight of these inducers (DPI7, DPI10, DPI12, DPI13, DPI17, DPI18, DPI19 and RSL3) can directly suppress GPx4 activity; however, similar to erastin, DPI2 does not affect GPx4 2. In addition, GPx4 knockdown of HT‐1080 cells with siRNAs, which lower the level of GPx4 mRNA by 20 times, can result in cell death and the accumulation of lipid peroxides in cells. Similar to RSL3‐induced ferroptosis, such cell death can be rescued with DFOM (an iron chelator), U0126 (a MEK inhibitor), and vitamin E (an antioxidant) 2, suggesting that GPx4 activity inhibition is a major contributor to ferroptosis. Moreover, GPx4 is currently believed to be a key target in ferroptosis triggered by a variety of ferroptosis inducers, including erastin and RSL3 2.

### Other mechanisms underlying ferroptosis

Voltage‐dependent anion channels (VDACs), also known as membrane porin protein, are the transmembrane channels for transporting ion and metabolites in eukaryotic cells 51, 52. Large amounts of VDACs are distributed on the mitochondrial outer membrane. Erastin can bind with VDAC2 and VDAC3 on the mitochondrial outer membrane to alter membrane permeability and slow down the oxidation of NADH. Moreover, erastin alters the ion selectivity of the channels and allows only cations to move into mitochondria 53, causing mitochondrial dysfunction and oxidant release that ultimately lead to oxidation‐dependent non‐apoptotic cell death, namely, ferroptosis 53. Recent research found that through the P53‐SLC7A11P53 axis, P53 could suppress SLC7A11 expression, thus impeding the uptake of cystine and promoting the occurrence of ferroptosis. The effect of P53 can be blocked by ferroptosis inhibitors rather than by the inhibitors specific for apoptosis, necrosis and autophagy. In addition, P53 increases the intracellular ROS level and triggers the ROS‐induced stress response, ultimately enhancing the susceptibility of tumour cells to ferroptosis 54, 55.

## Regulatory pathways of ferroptosis

### The MVA pathway

The MVA pathway is an important contributor to selenoprotein synthesis, and GPx4 is a selenoprotein with selenocysteine in its active centre. However, because the genetic code of selenocysteine is UGA, which is identical to the termination codon, a specific transporter is required for the insertion of selenocysteine into GPx4 56. This transporter is selenocysteine tRNA, which contains isopentenyladenosine and is capable of decoding the genetic code of selenocysteine and precisely inserting selenocysteine into the corresponding protein. However, the maturation of selenocysteine tRNA requires tRNA‐isopentenyl transferase to catalyse the transfer of the isopentene group of isopentenylpyrophosphate (IPP) to the specific adenine sites of selenocysteine tRNA precursors 57. Because IPP is an important product of the MVA pathway, MVA pathway inhibitors (e.g. statins) can impede selenocysteine tRNA maturation and GPx4 synthesis 5, 57.

### The sulphur‐transfer pathway

Sulphur‐containing proteins are very important in mammal cells. Methionineis a sulphur‐containing amino acid that is essential for human bodies and can only be obtained from food. Through the sulphur‐transfer pathway in the body, methionine can be converted to S‐adenosyl homocysteine and cysteine. Under conditions of cysteine insufficiency, homocysteine is converted into cystathionine (a precursor of cysteine) to ultimately supplement the cysteine pool through the sulphur‐transfer pathway 58. Various studies have demonstrated that the cysteine in more than 40% of the sulphur‐containing amino acids in mammals comes from their food 59, 60. The cysteine in bodies is mostly used to synthesize GSH, anti‐oxidative peptides, thioredoxin (Trx), etc.; of these, GSH is a pivotal factor for maintaining the cellular oxidation‐reduction balance. GSH mediates the reduction of the lipid peroxides and organic hydroperoxide products of alcohols in cells *via* GPx4; thus, it enables GPx4 to play a central regulatory role in ferroptosis 2. Under oxidative stress conditions, cystathionine‐β‐synthetase activation promotes methionine‐to‐cysteine conversion and GSH synthesis through the sulphur‐transfer pathways 58, thus protecting cells from the injury caused by the oxidative stress response. Recent research revealed that by upregulating the gene expression of the sulphur‐transfer pathways, the loss of cysteinyl‐tRNA synthetase (CARS) could inhibit erastin‐induced ferroptosis but not RSL3‐ or BSO‐induced ferroptosis 61. This suggests that sulphur‐transfer pathways play a negative regulatory role in cell ferroptosis.

### The HSF1‐HSPB1pathway

Heat‐shock proteins (HSPs) have been considered a molecular partner that regulates and controls the construction of the cellular skeleton 62 and stabilizes abnormally folded proteins 63. There are six HSP families: HSP100, HSP90, HSP70, HSP60, HSP40 and small HSPs. Heat‐shock factors (HSFs) are the transcription factors that regulate HSP synthesis 64. HSPB1 is also known as mouse HSP25 or human HSP27. Recent research has found that erastin could enhance the expression of HSPB1 mRNA and protein and that the HSF1‐HSPB1 pathway could negatively regulate erastin‐induced ferroptosis in human cervical cancer cells, osteosarcoma cells and prostate cancer cells 65. HSF1 and HSPB1 inhibition increases the concentrations of iron and ROS in cells, ultimately suppressing the growth of tumour cells, whereas PKC‐regulated HSPB1 phosphorylation can prevent cell ferroptosis because phosphorylated HSPB1 inhibits the uptake of iron and lipid ROS by cells 65.

### Other regulatory pathways of ferroptosis

**Figure 1:**
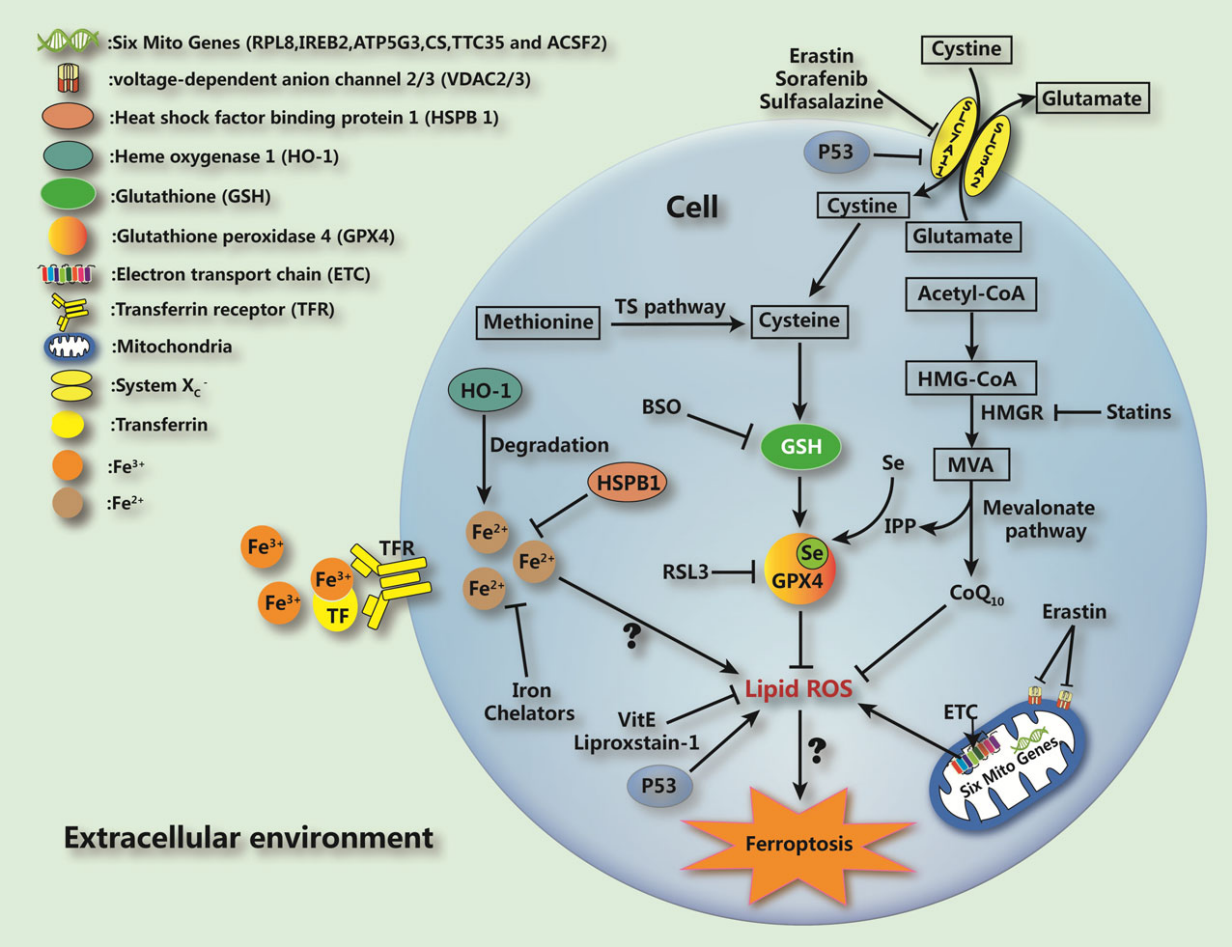
The occurrence and regulatory mechanisms of ferroptosis in a cell. Ferroptosis inducers such as erastin, sorafenib and sulfasalazine inhibit SLC7A11 insystemX_c_^−^ andimpede the uptake of cystine by cells, thus leading to a decline in intracellular cysteine and a subsequent reduction in glutathione (GSH), which requires cysteine for its synthesis; this ultimately results in declination of anti‐oxidative ability of cells. Through the sulphur‐transfer pathways, cellular methionine can be used to supplement the cysteine level. As a key component in ferroptosis, GPx4 can bind with GSH and suppress cellular lipid peroxides to prevent cellular ferroptosis. Class 2 ferroptosis inducers such as RSL3 can directly suppress GPx4 to induce ferroptosis. The MVA pathway plays an important role in regulating GPx4 maturation. Iron, which is indispensable to ferroptosis, can be transported from outside to the inside of the cells by transferrin. Ironchelators can impede ferroptosis. Mitochondria are the most important organelle involved in ferroptosis; they contain six ferroptosis‐related genes and release ferroptosis‐inducing lipid peroxides through the electron transport chain. In addition, a number of intracellular molecules/proteinscan regulate ferroptosis;e.g. P53 inhibits SLC7A11 and promotes the production of lipid peroxides. HSPB1 can inhibit ferroptosis by impeding the increase in intracellular iron. Some anti‐oxidants, such as vitamin E, liproxstain‐1 and ubiquinone (Co Q_10_), can impede ferroptosis by directly suppressing lipid peroxides.

Other pathways are also involved in the regulation of cellular ferroptosis. For example, glutamate and transferrin can regulate cell ferroptosis *via* the glutamine decomposition pathway and the transferrin receptors on the surface of cells 66, 67. Sun *et al*. found that the p62‐Keap1‐NRF2 pathway regulates the susceptibility of liver cancer cells to ferroptosis by regulating the expression of NRF2 68. In a recent study, Hasegawa *et al*. found that the MUC1‐C/xCT pathway could play a negative regulatory role and inhibit the erastin‐induced ferroptosis of triple‐negative breast cancer (TNBC) cells 69. Haem oxygenase‐1 (HO‐1) is an important source of intracellular iron, and Kwon *et al*. confirmed its pivotal role in erastin‐induced ferroptotic cell death, as evidenced by its ability to induce the lipid peroxidation reaction and cause cellular ferroptosis 70 (Fig. 1).

## Ferroptosis and tumours

In recent years, an increasing number of studies has revealed the close relationships of ferroptosis with various human diseases, including Huntington's disease (HD), periventricular leukomalacia (PVL) and renal functional damage 71, 72, 73. In addition, a number of tumour cells, such as diffuse large B‐cell lymphoma (DLBCL), RCC, liver cancer, cervical carcinoma, osteosarcoma and prostate adenocarcinoma cells 2, 65, are very susceptible to ferroptosis. However, the roles of ferroptosis in tumour occurrence, progression and treatment remain to be clarified. Various studies have confirmed the pivotal role of ferroptosis in killing tumour cells and suppressing tumour growth. The ferroptosis inducer erastin can improve the efficacy of chemotherapy when it is administered jointly with chemotherapeutic drugs such as temozolomide, cisplatin, cytarabine/ara‐C and doxorubicin/Adriamycin 74, 75. In tumour xenograft models, erastin, piperazine erastin and RSL3 impede the growth of tumours 2, 65. In addition, artemisinin derivatives can induce iron‐dependent cell death, particularly ferroptosis, suggesting that they can be used to treat ferroptosis‐susceptible tumours 4, 6.

### Diffuse large B‐cell lymphoma

In a study investigating the effects of erastin on tumour cells of various tissues (including hematopoietic cells, lymphatic tissue, and lung, large intestine, ovary and skin tissue), the researchers found that DLBCL cells were remarkably more sensitive to erastin than any other types of tumour cells 2. This increased sensitivity might be attributable to a deficiency of the sulphur‐transfer pathways in some types of leukaemia and lymphoma 76, 77. Such a deficiency results in an enhanced dependency of tumour cells on extracellular cysteine and cystine, anderastin inhibits the system X_C_^−^ mediated cystine uptake of the cells from the extracellular space. As a result, DLBCL cells are much more susceptible than other tumours to erastin‐induced ferroptosis. Further experiments revealed that compared with other hematopoietic tumours, DLBCL cells are particularly susceptible to erastin‐induced ferroptosis 2. In addition, SAS, which is a clinical drug and a ferroptosis inducer, can effectively inhibit the growth of DLBCL by suppressing the expression of SLC7A11 (a component of system X_C_^−^) 78, further indicating the important role of ferroptosis in the regulation of DLBCL growth. Both erastin and RSL3 can facilitate the production of lipid peroxides in two DLBCL cell lines, SU‐DHL‐8 and WSU‐SLCL‐2, and the ferroptosis induced by erastin in the two cell lines can be rescued by the antioxidant vitamin E, suggesting that the cell death is the result of ROS‐dependent ferroptosis 2.

### Hepatocellular carcinoma

HCC is the most common type of liver cancer; it is ranked fifth in prevalence and third in mortality among males worldwide 79. Currently, the treatment methods for liver cancer include surgical and non‐surgical treatments, but neither result in satisfactory outcomes, particularly for advanced liver cancer. Sorafenib, a multi‐kinase inhibitor, is the first drug to be used for the systematic treatment of advanced HCC and can significantly prolong the survival of HCC patients. A survey showed that approximately 40% of newly diagnosed HCC patients considered sorafenib the first choice for treatment 80. In an HCC cell line, treatment with deferoxamine (DFX), an iron chelator, remarkably reduced the toxicity of sorafenib, and this inhibitory effect could be reversed by lipophilic anti‐oxidants 15. In summary, ferroptosis can occur in liver cancer cells, and it can be induced and activated by sorafenib 3, 81.

During the ferroptosis of liver cancer cells, the p62‐Keap1‐NRF2 pathway plays a pivotal role. P62 inhibits the degradation of NRF2 by disrupting Keap1 and thus results in the accumulation of NRF2 in cells. NRF2 up regulates the expression of genes related to iron and ROS metabolism, including quinone oxidoreductase‐1 (NQO1), heme oxygenase‐1 (HO‐1), and ferritin heavy chain 1 (FTH1). Because all of these genes inhibit ferroptosis, NRF2 is a negative regulator of ferroptosis in liver cancer cells. *In vivo* and *in vitro* experiments using HCC cell lines showed that the inhibition of NRF2 expression *via* genetic tools or drugs could significantly enhance the anti‐tumour effects of erastin and sorafenib, whereas the activation of NRF2 expression led to cellular resistance to ferroptosis 68.

Retinoblastoma (RB) protein, a member of the protein family that regulates the transcriptional function of various genes in eukaryotic cells 82, is closely related to liver tumorigenesis and the ferroptosis of liver cancer cells. Studies using mouse models revealed a direct relationship between RB protein dysfunction and the occurrence of liver tumours 83, 84. Functional deficiencies of RB protein are common in human HCC cells. Previous research demonstrated that when exposed to sorafenib, HCC cells with decreased RB protein expression level had a death rate 2–3 times higher than that of cells with a normal RB protein expression level, indicating that HCC cells with a low RB protein level were more susceptible to ferroptosis 7. Furthermore, the researchers found that HCC cells with a decreased RB protein expression level suffered increased cytotoxicity when exposed to sorafenib, and moreover, the deactivation of RB protein enhanced the oxidative stress response of cells by increasing the production of reactive oxygen in mitochondria 7. These findings suggest that RB protein plays a critical role in regulating ferroptosis in liver cancer cells. According to existing studies, the sensitivity to sorafenib varies greatly among individuals 85, 86, and treatment with sorafenib alone cannot achieve satisfactory outcome. A study showed that the joint use of sorafenib and other kinase‐targeting compounds improved the anti‐proliferation effect and treatment efficacy of sorafenib 87. A recent study also found that the serum concentration of the oxidative stress response marker was correlated with the progression‐free survival duration in a portion of HCC patients undergoing sorafenib treatment 88, suggesting the pivotal role of sorafenib‐induced ferroptosis in the survival of HCC patients. Therefore, ferroptosis could be a new strategy for HCC treatment, and the above‐discussed NRF2 and RB protein could be important treatment targets in the future.

### Renal cell carcinoma

Research on the effect of erastin in 60 tumour cell lines of eight tissues found that RCC cells were more susceptible than others to erastin‐induced cell death. Further research confirmed that erastin could induce the death of RCC cells in a manner that has the general characteristic features of ferroptosis (namely, elevated lipid ROS production and decreased GPx4 expression) and can be inhibited with anti‐oxidants 2. In addition, because sorafenib is also a ferroptosis inducer, its clinical effectiveness in RCC treatment 89 indirectly supports the existence of ferroptosis in RCC.

### Pancreatic carcinoma

Pancreatic carcinoma is a highly fatal tumour, and even standardized pharmacotherapy can only prolong patients’ survival duration by less than 6 months 90. Artesunate (ART) and erastin can induce iron‐ and ROS‐dependent cell death, respectively (namely, ferroptosis) in ductal pancreatic cancer. In particular, ductal pancreatic cancer with a mutant KRas gene is more susceptible to ferroptosis. This type of cell death can be completely blocked by Ferr‐1 but cannot be inhibited by Nec‐1s, an inhibitor of apoptotic necrosis 6. In addition, the expression of xCT (SLC7A11) protein is up‐regulated in human pancreatic cancer cells, suggesting that ferroptosis might be related to the tumorigenesis of pancreatic carcinoma 38.

### Ovarian cancer

Ovarian cancer is the most common fatal tumour in women. ART, a derivative of artemisinin, can suppress the proliferation of ovarian cancer cells. ART‐treated ovarian cancer cells have a higher ROS production level, and the amount of ROS produced is ART‐dependent, leading to ROS‐dependent DNA damage and cell death. Moreover, transferrin pretreatment of ovarian cancer cells increases the intracellular iron level and enhances the sensitivity of the cells to ART, suggesting that iron has an important role in ART‐regulated cell death in ovarian cancer. The ferroptosis inhibitor ferrostatin‐1 can remarkably suppress ART‐induced cell death in ovarian cancer 91. All of these findings suggest that ART can induce their iron‐ and ROS‐associated cell death (i.e. ferroptosis) of ovarian cancer cells.

### Other ferroptosis‐related tumours

Ferroptosis can occur in rhabdomyosarcoma cells, as demonstrated by an experimental observation that erastin and RSL3 treatments can induce ferroptosis in these cells 92. Sun *et al*. found that HSPB1 can affect the erastin‐induced ferroptosis of human cervical cancer cells, prostate cancer cells and osteosarcomacells 65, suggesting a close correlation of ferroptosis with these tumourous diseases. Recent research found that the mucin 1 C terminal subunit (MUCI‐C)/xCT pathway in TNBC cells could inhibit the erastin‐induced ferroptosis of these cells, possibly because MUC1‐C can keep xCT (SLC7A11) stable 69 and thus suppress the erastin‐caused inhibition of SLC7A11.

## Summary and outlook

Regarding ferroptosis, a newly discovered cell death form, a variety of questions remain despite increasing research progress in understanding its induction mechanism and signalling pathway. GPx4 is believed to be an important core molecule that regulates the ferroptosis process, but the role of iron and the mechanism underlying the effect of iron in ferroptosis remain unclarified. Clarification of the definitive role of iron in ferroptosis will be very helpful to our understanding of the occurrence and regulatory mechanisms of ferroptosis.

Although research has revealed that ferroptosis is related to various diseases, the role of ferroptosis in human diseases remains a mystery. The susceptibility of cells to ferroptosis varies greatly among different tissues, and the sensitivity to ferroptosis inducers (e.g. sorafenib) differs significantly among individuals. Therefore, identifying an indicator that can reflect the susceptibility of cells and individuals is of great significance for improving our understanding, diagnosis, and treatment of ferroptosis‐related diseases. We believe that in the near future, ferroptosis will become a new strategy for treating tumourous diseases that currently have no successful treatments.

## Conflicts of interest

The authors confirm that there are no conflicts of interest.
